# Pathogenicity of monokaryotic and dikaryotic mycelia of *Ganoderma boninense* revealed via LC–MS-based metabolomics

**DOI:** 10.1038/s41598-024-56129-8

**Published:** 2024-03-04

**Authors:** Krystle Angelique A. Santiago, Wei Chee Wong, You Keng Goh, Seng Heng Tey, Adeline Su Yien Ting

**Affiliations:** 1 School of Science, Monash University Malaysia, Jalan Lagoon Selatan, 47500 Bandar Sunway, Selangor Darul Ehsan Malaysia; 2 Advanced Agriecological Research Sdn. Bhd., 11 Jalan Teknologi 3/6, Taman Sains Selangor 1, Kota Damansara, 47810 Petaling Jaya, Selangor Darul Ehsan Malaysia

**Keywords:** Amino acid metabolism, Dikaryon, Functional activities, Monokaryon, Oil palm, Applied microbiology, Environmental microbiology, Fungi

## Abstract

This study compared the pathogenicity of monokaryotic (monokaryon) and dikaryotic (dikaryon) mycelia of the oil palm pathogen *Ganoderma boninense* via metabolomics approach. Ethyl acetate crude extracts of monokaryon and dikaryon were analysed by liquid chromatography quadrupole/time-of-flight-mass spectrometry (LC-Q/TOF–MS) coupled with multivariate data analysis using MetaboAnalyst. The *mummichog* algorithm was also used to identify the functional activities of monokaryon and dikaryon without a priori identification of all their secondary metabolites. Results revealed that monokaryon produced lesser fungal metabolites than dikaryon, suggesting that monokaryon had a lower possibility of inducing plant infection. These findings were further supported by the identified functional activities. Monokaryon exhibits tyrosine, phenylalanine, and tryptophan metabolism, which are important for fungal growth and development and to produce toxin precursors. In contrast, dikaryon exhibits the metabolism of cysteine and methionine, arginine and proline, and phenylalanine, which are important for fungal growth, development, virulence, and pathogenicity. As such, monokaryon is rendered non-pathogenic as it produces growth metabolites and toxin precursors, whereas dikaryon is pathogenic as it produces metabolites that are involved in fungal growth and pathogenicity. The LC–MS-based metabolomics approach contributes significantly to our understanding of the pathogenesis of *Ganoderma boninense*, which is essential for disease management in oil palm plantations.

## Introduction

Fungal pathogenicity is often linked to the synthesis of secondary metabolites during pathogenesis^1,2^, as well as their life cycle and morphological development^3^. Some fungal pathogens such as *Zymoseptoria tritici* (wheat pathogen) exhibits a complex life cycle that involves various morphotypes that are essential to pathogen survival and disease epidemiology^3^. The non-infectious chlamydospores of *Z. tritici* are produced to resist environmental stress, and they later develop into the infectious ascospores that infect wheat. These ascospores germinate to produce non-infectious hyphae that penetrate and colonize the plant, which is then followed by the formation of the infectious pycnidiospores that cause secondary infections in wheat^3^. Fungi can also switch between unicellular yeast and multicellular filamentous growth (i.e., dimorphism) as part of their life cycle^4^. The dimorphic fungus *Ustilago maydis,* for example, is a biotrophic pathogen of corn that exhibits the haploid saprophytic yeast form to support fungal growth and development, and mycelial dikaryotic stage to invade and infect the plant^5^.

In Malaysia, the plant pathogen *Ganoderma boninense* is known to infect oil palm trees (*Elaeis guineensis* Jacq.), one of the most valuable commercial crops in the country^6^. Malaysia has the second largest oil palm plantations (about 5.8 million hectares) worldwide and produces more than 19.5 million tons of palm oil, which contribute approximately USD 16 billion to Malaysia export revenue^7^. *G. boninense* is a hemibiotroph that causes the basal stem rot (BSR) disease of oil palm that is characterized by the gradual decay of the roots, bole and trunk tissues of the oil palm, which then progresses to palm toppling^8,9^. It was estimated that *G. boninense* may infect 860,610 ha of mature oil palms by 2040, adversely impacting the economy^9^. *G. boninense*, therefore, is an economically devastating pathogen that must be controlled.

*Ganoderma boninense* is known to undergo a complex life cycle that includes the monokaryotic and dikaryotic stages^9^. The monokaryotic stage involves the formation of vegetative and non-infectious mycelium (monokaryon) from basidiospores, followed by the dikaryotic stage where fusion of compatible mating types takes place to form the infectious dikaryotic mycelium (dikaryon)^9^. The resulting dikaryon colonizes and infects the roots and basal stem of the oil palm^6,9^. The pathogenicity of dikaryotic *G. boninense* to oil palm is established, typically through pathogenicity assays performed using both monokaryotic and dikaryotic mycelia of *G. boninense* on germinated seeds and oil palm seedlings^6^. In these studies, physical changes and symptom development in oil palm seedlings are often indicators of the pathogenicity of the tested monokaryotic and dikaryotic mycelia. There is, however, no current information on the pathogenicity of monokaryotic and dikaryotic mycelia of *G. boninense* at the metabolic level. Therefore, the metabolites that may contribute to pathogen virulence and pathogenesis are not well understood. Additionally, the identification of both non-infective and infective and highly pathogenic mycelia of *G. boninense* is crucial to the development of disease management in oil palm plantations. For example, fungicides that selectively target monokaryotic mycelia can be developed to prevent its development into dikaryotic mycelia, thereby intervening the infection process. Similarly, fungicides and cultural practices that target dikaryotic mycelia can be developed to reduce the effects and spread of infection caused by *G. boninense* to oil palm.

In this study, the metabolic profiles of monokaryotic (monokaryon) and dikaryotic (dikaryon) mycelia of *G. boninense* are compared using LC–MS-based metabolomics. Metabolomics is a powerful tool that enables comprehensive and large-scale analysis of metabolites in any biological sample, which are mainly based on mass spectrometry and nuclear magnetic resonance^10,11^. However, the functional interpretation of high throughput metabolomics by mass spectrometry is hindered by the need for secondary metabolites to be identified^10^. Interestingly, the functional activities can be predicted directly from mass spectrometry data without a priori identification of metabolites by incorporating *mummichog* algorithm in the analyses^10^. For this study, the metabolite profile of dikaryon has been reported in an earlier study^12^ and is used in this study to compare with the metabolite profile of monokaryon. The functional activities interpreted from the extracted secondary metabolites were determined using *mummichog* algorithm via the platform MetaboAnalyst ver. 5.0^13^. These functional activities explained the non-infectious and infectious profiles of monokaryon and dikaryon, respectively. The results of this study, as well as the tools used, can provide insights into the mechanisms by which *G. boninense* causes disease to oil palm. Additionally, the results can aid in identifying important genes that enable oil palm to resist the pathogen. This approach can also be used as a model to profile metabolic differences between the monokaryotic and dikaryotic mycelia of other economically important fungal plant pathogens, to provide more information on their pathogenicity and their disease management.

## Materials and methods

### Culture establishment and preparation of oil palm extract medium (OPEM) broth

Monokaryotic (Strain 2001A/4F1/1) and dikaryotic (GenBank accession number MN490049) mycelia of *Ganoderma boninense* were obtained as pure cultures from Advanced Agriecological Research Sdn. Bhd. The cultures were inoculated onto malt extract agar (MEA, Difco™, Becton Dickinson and Company, USA) and incubated at 28 °C in the dark for 18 and 15 days, respectively. Following incubation, eight mycelial plugs (8 mm in diameter) of each monokaryotic (monokaryon, mGb) and dikaryotic (dikaryon, dGb) *G. boninense* were inoculated into 1000 ml Erlenmeyer flask containing 600 ml of oil palm extract medium (OPEM) broth. The oil palm extract medium (OPEM) broth was prepared by blending 150 g of oil palm sawdust obtained from healthy palm with 1000 ml of distilled water^12^. The plant collection (oil palm trunk tissues) and use were in accordance with all relevant guidelines. OPEM stimulates the growth of *G. boninense* as the natural components of the oil palm were provided. The resulting broth was squeezed and filtered through a cheesecloth. The OPEM were autoclaved using standard settings (121 °C, 15 min, 15 psi). The monokaryon-inoculated OPEM (OPEM + mGb) and dikaryon-inoculated OPEM (OPEM + dGb) cultures were incubated at room temperature (26 ± 2 °C) with agitation (120 rpm) at different sampling time points (Day 1, 3, 7, 14, 21 and 28). The sampling performed at various time points allowed for the detection of the metabolite production of *G. boninense* during the early oil palm-*Ganoderma* interaction*.* Three biological and three technical replicates for both monokaryon and dikaryon were prepared for each time point. Uninoculated OPEM served as the negative control and was also prepared in triplicates.

### Extraction of secondary metabolites produced by monokaryon and dikaryon

The secondary metabolites produced by both monokaryon (OPEM + mGb) and dikaryon (OPEM + dGb), as well as those found in the negative control (OPEM), were extracted via liquid–liquid extraction^12^. Each broth culture (600 ml) was filtered using a sterile miracloth (Merck, Darmstadt, Germany) to separate the mycelia from the broth and other oil palm debris. The filtered broth collected was extracted using the solvent ethyl acetate (analytical grade, Systerm Chemicals, Malaysia) by adding equal volume as the broth. The mixture was then poured into a separating funnel, sealed with a glass cork and shaken vigorously. The mixture was left for 5 min or until two layers (organic and aqueous layers) were formed. The organic solvent with the dissolved secondary metabolites was collected and dried using a rotary evaporator (Buchi Rotavapor R-3, Buchi, Switzerland). The solvent collected from the rotary evaporator was again mixed with the aqueous layer (i.e., broth) and the extraction was repeated. The resulting crude extracts were collected in pre-weighed vials, covered with sterile miracloth and were left undisturbed for one day in a fumehood until the solvent evaporated completely. All dried crude extracts were kept at − 80 °C until further use.

### Metabolite profiling of crude extracts of monokaryon and dikaryon using liquid chromatography quadrupole/time-of-flight-mass spectrometry (LC-Q/TOF–MS) analyses

High-resolution LC–MS (LC-HRMS) was carried out as described by Santiago et al.^12^. Briefly, a concentration of 1 mg/ml of each crude extract (OPEM + mGb, OPEM + dGb and OPEM) was prepared. The samples were analyzed using the Agilent 1290 Infinity LC system coupled to the Agilent 6520 Accurate-Mass Q-TOF mass spectrometer (Agilent, California, USA) with electrospray ionization (ESI) interface in negative ion mode equipped with an Agilent Eclipse XDB-C18 column (150 mm × 2.1 mm column, Agilent, California, USA). The column conditions were as described by Santiago et al.^12^. The mobile phase consisted of 0.1% formic acid in water and 0.1% formic acid in acetonitrile. The run time for the analysis was 25 min followed by a 5-min post-run time to minimize carry-over between injections. The collected data were processed using the Agilent MassHunter Qualitative Analysis B.07.00 (Agilent, California, USA). The secondary metabolites were identified by comparing with those recorded in the METLIN database. Three technical replicates were used in this study.

### Metabolomics analyses of the crude extracts of monokaryotic and dikaryotic *G. boninense*

The raw LC–MS data of monokaryon (OPEM + mGb) and dikaryon (OPEM + dGb) crude extracts were initially converted to mzML as described by Santiago et al.^12^. The processed data were then exported as CSV files containing information of the detected peaks, retention time and *m/z* values, to MetaboAnalyst ver. 5.0^13^. Data were normalized (normalization by sum), transformed (log transformation), scaled (Pareto scaling) and were further analyzed using principal component analysis (PCA) and orthogonal partial least-squares discriminant analysis (OPLS-DA). For the PCA, five components were analyzed to explain the variance between OPEM + mGb and OPEM + dGb crude extracts. The OPLS-DA, on the other hand, was used to assess the significant difference between secondary metabolites extracted from the OPEM + mGb and OPEM + dGb crude extracts. Cross-validation (CV) was performed using a tenfold CV method indicating the accuracy, Q_2_ and R_2_ values. A permutation test was also done to validate the model with the permutation *p-*value of *p* < 0.01.

To predict the functional activities of the extracted secondary metabolites, a functional pathway analysis was performed using the MS Peaks to Pathways module^10^ in MetaboAnalyst ver. 5.0^13^. A two-column spectral feature table containing *m/z* and *p-*values of the detected secondary metabolites was prepared separately for OPEM + mGb and OPEM + dGb crude extracts. Both spectral feature tables were exported as CSV files. The *mummichog* algorithm^10^ was then applied to leverage the power of known metabolic pathways to gain functional insight from the uploaded spectral feature table. The *p-*value cutoff was 0.00001. The metabolic model used for this analysis was *Saccharomyces cerevisiae* with data derived from the Kyoto Encyclopedia of Genes and Genomes (KEGG) library. The functional pathway analysis was graphically represented as a scatter plot, where the color and size of each circle corresponds to its *p*-value and enrichment factor, respectively. A darker color indicates a lower *p-*value (i.e., significant) and a larger circle indicates a higher number of compound hits in a pathway (i.e., well-represented pathway). The enrichment factor of a pathway is calculated as the ratio between the number of significant pathway hits and the expected number of compound hits within the pathway^14^. In addition to the scatter plot, a table containing ranked pathways, compound hits, total number of hits per pathway and *p*-values was generated. Once the functional activities and their respective significant metabolites are identified, a Metabolite Set Enrichment Analysis (MSEA) was also performed using MetaboAnalyst ver. 5.0 to determine the false discovery rate (FDR) of each identified functional activity to evaluate the proportion of false positives among the claimed positives^14^.

## Results

### Metabolite profiling of monokaryotic and dikaryotic mycelia of *G. boninense*

The LC–MS analyses of crude extracts of monokaryotic *G. boninense* (OPEM + mGb) revealed 12 secondary metabolites via the negative ionization mode (Supplementary Material Table S1). OPEM + mGb extracts had alkaloids (e.g., theobromine, 5-methoxycanthin-6-one), hydroxybenzoic acid (e.g., p-salicylic acid), flavonoids (e.g., 5,7,3′-trihydroxy-3,6,8,4′,5′-pentamethoxyflavone), sesquiterpene lactone (e.g., ligulatin B), glycosides (e.g., torachrysone 8-(6-oxalylglucoside), ferulic acid 4-O-glucuronide), flavone (e.g., carpelastofuran), cyclic dicarboxylic anhydride (e.g., 14-dihydroxycornestin) and long-chain fatty acids (e.g., 5,8,12-trihydroxy-9-octadecenoic acid, 11-hydroperoxy-12,13-epoxy-9-octadecenoic acid) (Supplementary Material Table S1). From the 12 compounds, 11 were naturally found in plants (e.g., alkaloids, hydroxybenzoic acid, flavonoids, glycosides, sesquiterpene lactone, long-chain fatty acids), while one was identified from fungi (e.g., cyclic dicarboxylic anhydride). It is presumed that the secondary metabolites such as torachrysone 8-(6-oxalylglucoside), 5,8,12-trihydroxy-9-octadecenoic acid, 11-hydroperoxy-12,13-epoxy-9-octadecenoic acid and theobromine may have originated from the uninoculated OPEM (control) as OPEM contains alkaloids, flavonoids, glycosides, peptides and other plant metabolites found in flowering trees and perennial herbs. In contrast, the crude extracts of dikaryotic *G. boninense* (OPEM + dGb) revealed a higher number of secondary metabolites (and higher fungal metabolites), which include aldehydes, fatty acids, glycosides, lactones, phenolic acids and sugars. From these 20 metabolites, 17 of the metabolites (e.g., aldehydes, glycosides, sugars, fatty acids) were plant metabolites and three (e.g., lactones, phenolic acids) were fungal metabolites. These findings suggested that dikaryon produced a higher number of fungal metabolites than monokaryon.

It was also observed that the secondary metabolites detected in OPEM + mGb crude extracts on Days 1, 3, 7, 14, 21 and 28 were naturally-occurring plant metabolites (e.g., 5,7,3′-trihydroxy-3,6,8,4′,5′-pentamethoxyflavone, 5,8,12-trihydroxy-9-octadecenoic acid, 11-hydroperoxy-12,13-epoxy-9-octadecenoic acid, ligulatin B, 5-methoxycanthin-6-one, scandenin) and may be derived from OPEM (Table 1). The fungal metabolite 14-dihydroxycornestin was only detected at Day 28 (Supplementary Material Table S1). Additionally, the metabolites p-salicylic acid and theobromine were only detected until Day 14, which may suggest that monokaryotic *G. boninense* was able to either utilize or degrade them. Similarly, the plant-derived metabolites detected in OPEM + dGb extracts were observed in all time points and may have originated from OPEM. The detection of fungal metabolites (e.g., aspulvinone H, bergenin and methylisocitric acid) in OPEM + dGb extracts, however, occurred as early as Day 14. These findings may suggest that monokaryon exhibited slower metabolism compared to dikaryon, resulting in lesser fungal metabolites produced. Additionally, monokaryon may have prioritized essential metabolism that is required for its growth and development over other metabolic activities such as synthesis of fungal metabolites that are necessary for plant colonization and pathogenesis. In contrast, dikaryon may have easily adapted to its environment, thereby requiring less growth metabolites. As such, dikaryon produced a higher number of fungal metabolites, which are responsible for infecting plants.

**Table 1:** Functional analysis of the secondary metabolites produced by the monokaryotic mycelia of *Ganoderma boninense.*

Pathway	Significant hits	− log10 *p-*value	FDR	Compound hits
Tyrosine metabolism	11	3.31599	4.56E−14	3-Methoxy-4-hydroxyphenylglycolaldehyde (C05583), 3,4-dihydroxymandelaldehyde (C05577), 3,4-dihydroxyphenylethyleneglycol (C05576), 3,4-dihydroxyphenylacetaldehyde (C04043), 4-hydroxyphenylacetaldehyde (C03765), 3-methoxy-4-hydroxymandelate (C05584), 3,4-dihydroxyphenylacetate (C01161), Homovanillate (C05582), 4-hydroxyphenylacetate (C00642)
Phenylalanine metabolism	4	1.724389	2.93E−03	Phenylacetaldehyde (C00601), phenylpyruvate (C00166), phenylacetic acid (C07086)
Phenylalanine, tyrosine and tryptophan metabolism	5	0.8760831	1.28E−02	Indoleglycerol phosphate (C03506), Phenylpyruvate (C00166), l-arogenate (C00826), 3-dehydroquinate (C00944), 3-(4-hydroxyphenyl) pyruvate (C01179)

Compound hits were based on Kyoto Encyclopedia of Genes and Genomes (KEGG) library. KEGG number is written in the parenthesis after the compound name.

### Metabolomics and functional analyses of the crude extracts of monokaryotic and dikaryotic *G. boninense*

**Figure 1:**
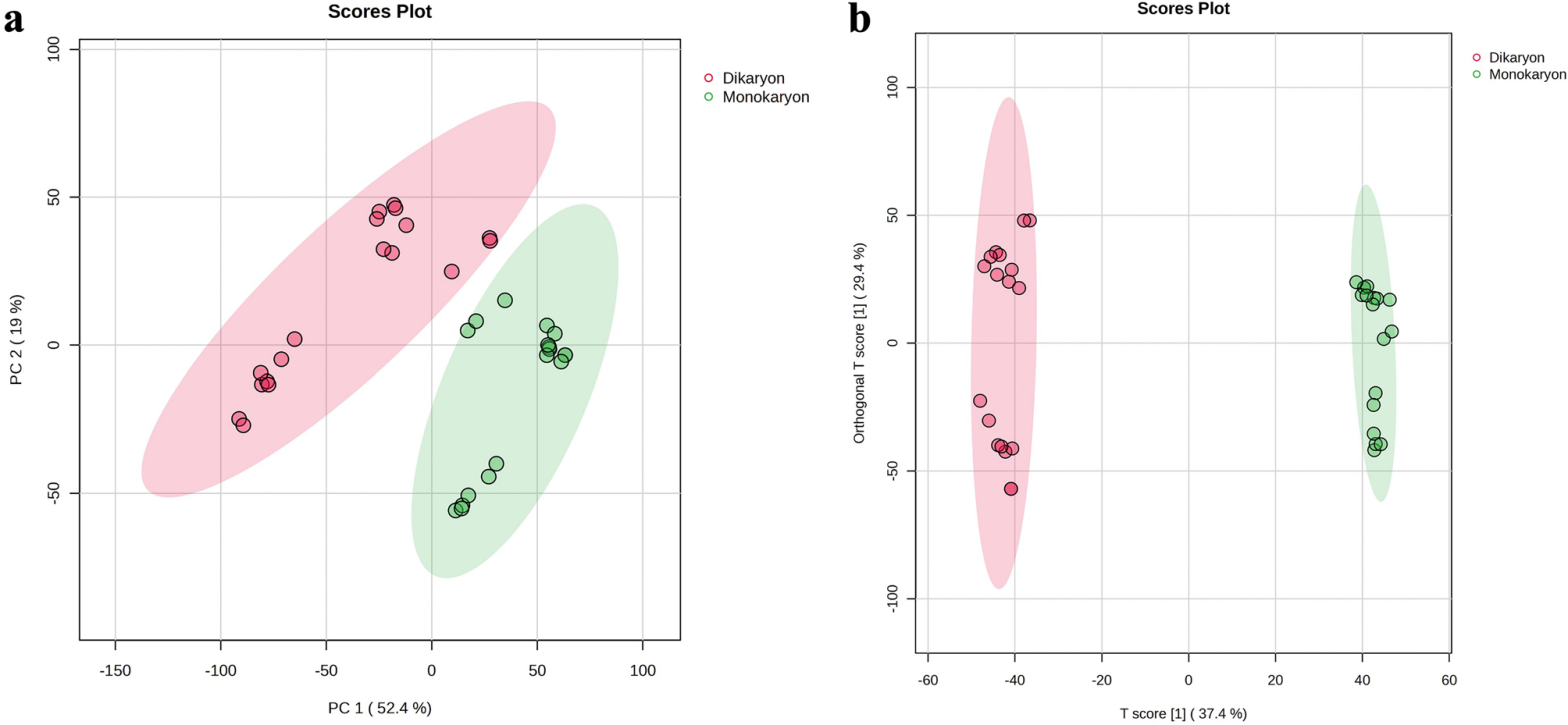
The multivariate data analyses of the crude extracts of the monokaryotic and dikaryotic *G. boninense* showing their (**a**) principal component analysis (PCA) and (**b**) orthogonal partial least-squares discriminant analysis (OPLS-DA) scores plots. The explained variances are shown in brackets.

The principal component analysis (PCA) showed distinct groupings between the monokaryotic (monokaryon) and dikaryotic (dikaryon) *G. boninense* (Fig. 1a). The five principal components (PC) used in generating the PCA gave a total explained variance of 82.8%. From these five PCs, the first two PCs (i.e., PC1 (52.4%) and PC2 (19%)) captured the most information from the given datasets (Fig. 1a), which led to a clear separation between monokaryon and dikaryon. To further explain the groupings in the PCA, an orthogonal partial least-squares discriminant analysis (OPLS-DA) was performed (Fig. 1b).

**Figure 2:**
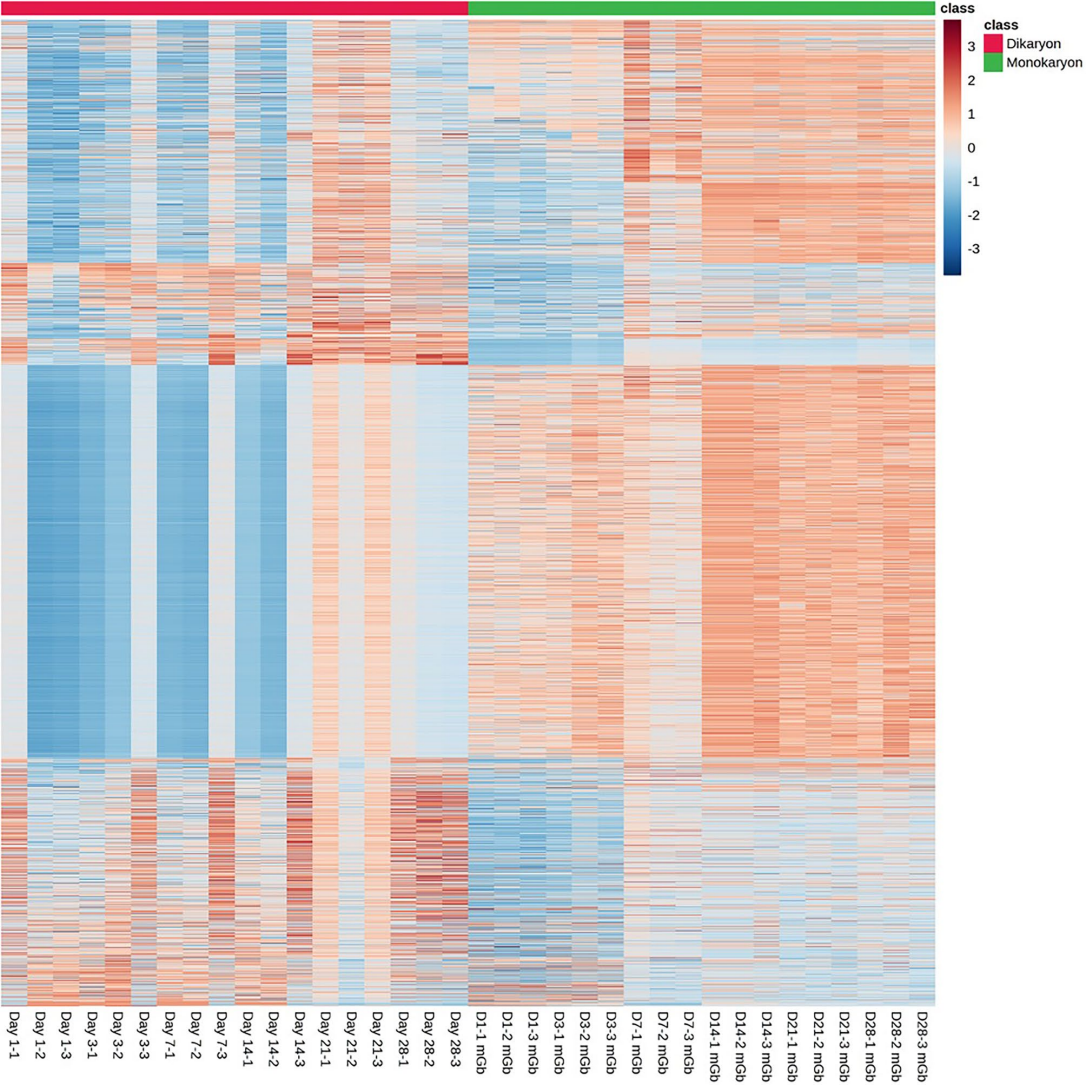
The heatmap of the monokaryotic and dikaryotic *G. boninense* for each time point (Days 1, 3, 7, 14, 21 and 28) ranked according to their t-test scores. The colored boxes on the right indicate the class used in this study, with the green box indicating monokaryon and the red box indicating dikaryon. The colors in the heatmap represent the relative concentration of each secondary metabolite in the sample, with an intense red color indicating high concentration of metabolite and an intense blue color indicating low concentration of metabolite under study. Samples labelled with “mGb” is used to differentiate from dikaryon samples.

The OPLS-DA score plot [Q2 = 0.982, R2Y = 0.996 (*p* < 0.01)] was used to categorize the detected secondary metabolites to either monokaryon or dikaryon, thereby accurately separating the two groups (Fig. 1b). The score plot had a T score of 37.4%, which indicates that 37.4% of the variance between monokaryon and dikaryon was explained (Fig. 1b). The score plot also discriminated secondary metabolites that separated monokaryon and dikaryon. Furthermore, the plot also showed an orthogonal T score of 29.4% indicating that 29.4% of the variations do not contribute to the separation of the groups, which reduced the “noise” in the model, thereby improving the accuracy of group separation (Fig. 1b). The group classification via PCA and OPLS-DA was further supported by the generation of heatmap, which showed the distinction between the metabolite profile of monokaryon and dikaryon (Fig. 2).

**Figure 3:**
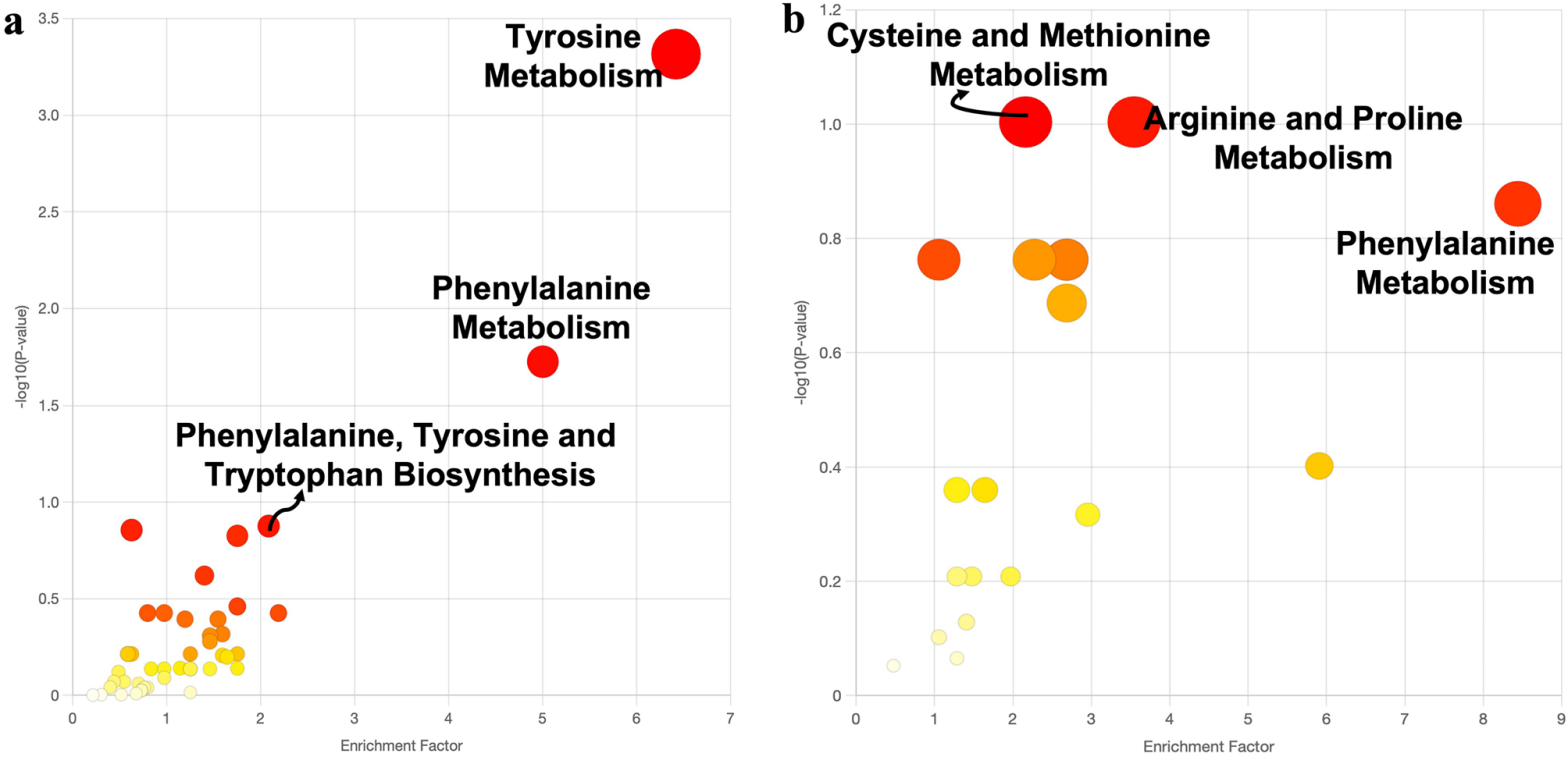
The summary of the functional activities of (**a**) monokaryotic and (**b**) dikaryotic *G. boninense* using *mummichog* algorithm graphically represented as a scatter plot. The color and size of each circle corresponds to its *p-*value and enrichment factor, respectively. A darker color indicates a lower *p-*value and a bigger circle indicates a higher number of compound hits in a pathway. The enrichment factor of a pathway is calculated as the ratio between the number of significant pathway hits and the expected number of compound hits within the pathway.

**Figure 4:**
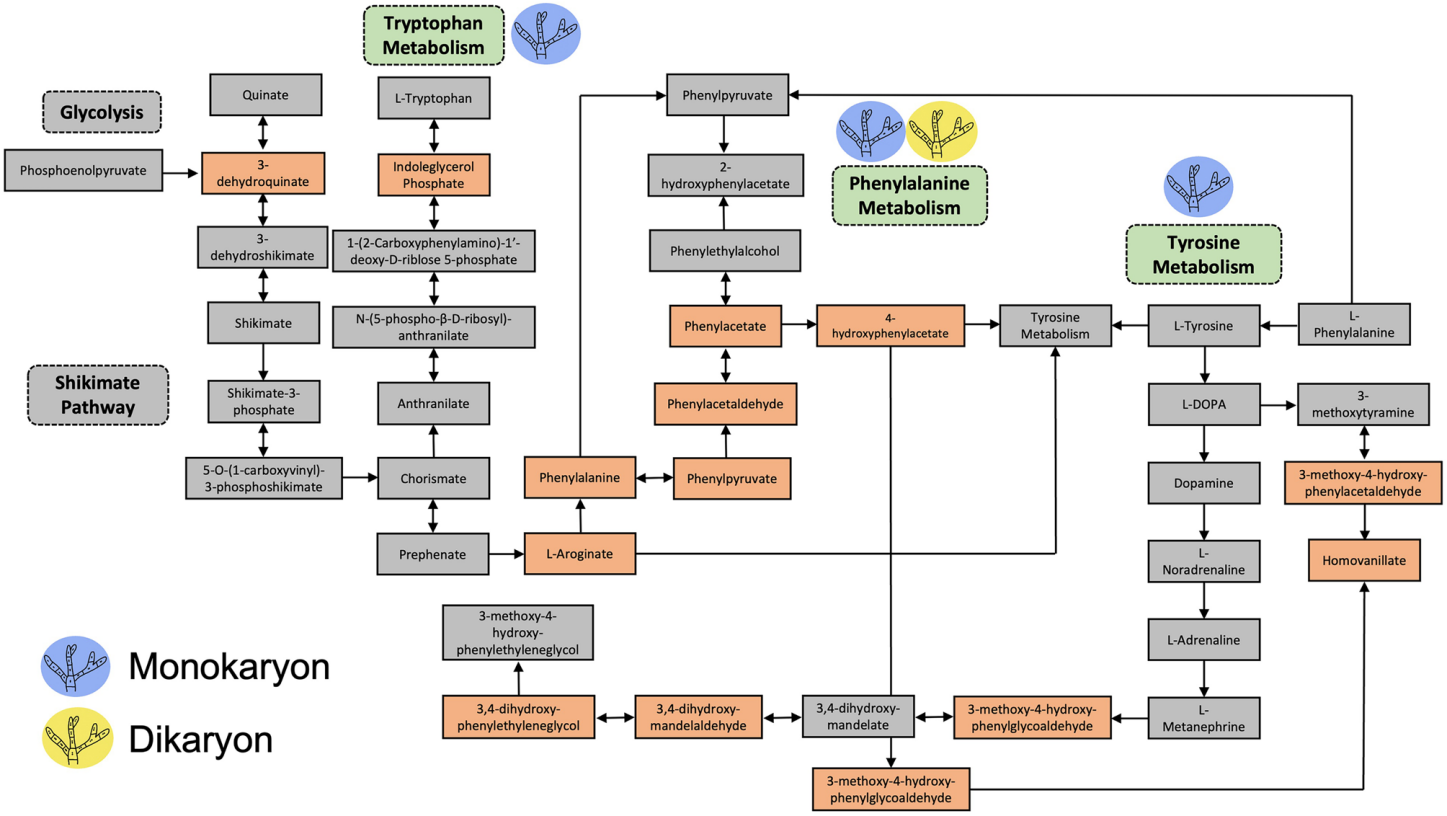
Simplified metabolic pathways showing the potential functional activities of monokaryotic and dikaryotic *G. boninense.* The identified functional activities (metabolism of tryptophan, tyrosine and phenylalanine) are shown in green dashed boxes. Other important processes and/or metabolic pathways not identified in this study are shown in gray dashed boxes. Significant secondary metabolites identified in this study for each metabolism are shown in orange boxes. Secondary metabolites in the gray boxes were not identified in this study but were included only to accurately illustrate the metabolic pathways. The metabolic pathways were adapted from Kyoto Encyclopedia of Genes and Genomes (KEGG) for the model organism *Saccharomyces cerevisiae.*

**Figure 5:**
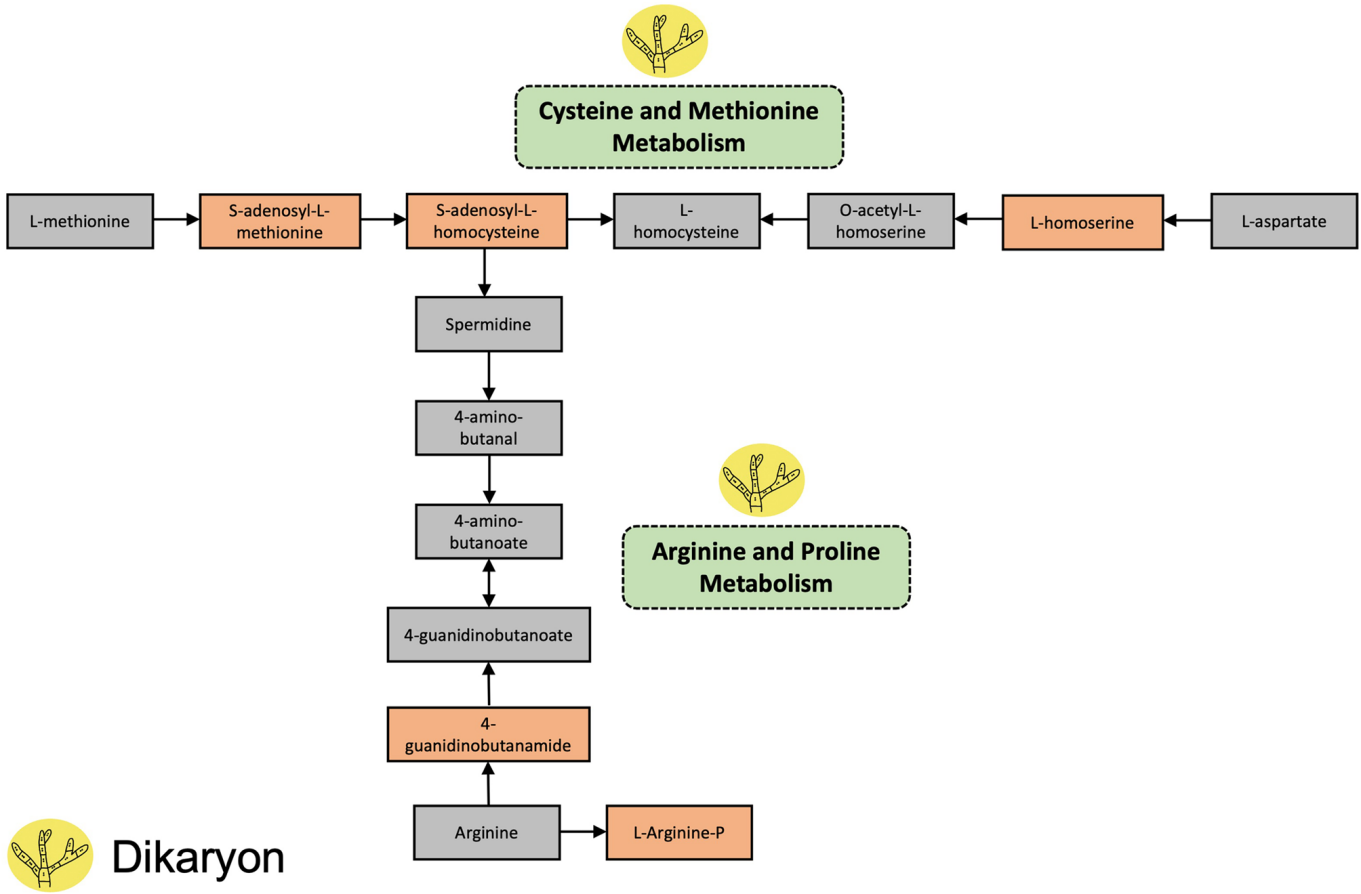
Simplified metabolic pathways showing the potential functional activities of monokaryotic and dikaryotic *G. boninense.* The identified functional activities (metabolism of cysteine and methionine, arginine and proline and phenylalanine) are shown in green dashed boxes. Significant secondary metabolites identified in this study for each metabolism are shown in orange boxes. Secondary metabolites in the gray boxes were not identified in this study but were included only to accurately illustrate the metabolic pathways. The metabolic pathways were adapted from Kyoto Encyclopedia of Genes and Genomes (KEGG) for the model organism *Saccharomyces cerevisiae.*

The functional activities of all the detected secondary metabolites (including known metabolites via METLIN database and unknown/unidentified metabolites) in the crude extracts were also compared to determine specific activities, such as metabolism, degradation or biosynthesis of secondary metabolites, that distinguish monokaryon from dikaryon (Fig. 3, Tables 1 and 2). In this study, the functional activities of the secondary metabolites produced by monokaryon include tyrosine metabolism, phenylalanine metabolism and the biosynthesis of phenylalanine, tyrosine and tryptophan (Fig. 3A, Table 1). These activities are involved in fungal growth and development and in the synthesis of toxin precursors. In this study, tyrosine metabolism is the most enriched metabolic pathway, followed by phenylalanine metabolism and phenylalanine, tyrosine and tryptophan biosynthesis (Fig. 3A). Tyrosine metabolism is represented by 3-methoxy-4-hydroxyphenylglycolaldehyde, 3,4-dihydroxymandelaldehyde, 3,4-dihydroxyphenylacetaldehyde, 3-methoxy-4-hydroxyphenylacetaldehyde, 4-hydroxypenylacetaldehyde, 3-methoxy-4-hydroxymandelate, 3,4-dihydroxyphenylacetate, homovanillate and 4-hydroxyphenylacetate, which provide a nitrogen source to the fungus (Fig. 4, Table 1). These secondary metabolites are significantly enriched, which suggest that monokaryon produced these metabolites in high concentrations relative to other metabolites present in the samples. In contrast, the functional activities of secondary metabolites produced by dikaryon include the metabolism of cysteine and methionine, arginine and proline and phenylalanine (Fig. 3b, Table 2). These functional activities are important in the physiology, virulence and pathogenicity of the fungus. For dikaryon, phenylalanine metabolism is the most enriched metabolic pathway, followed by arginine and proline metabolism and cysteine and methionine metabolism (Fig. 3b). Phenylalanine metabolism is represented by the metabolites phenylpyruvate and phenylacetic acid, which are involved in fungal growth and development and the production of other secondary metabolites (Fig. 5, Table 2). In short, the identified functional activities and metabolic pathways showed similarities and differences between the monokaryotic and dikaryotic mycelia of *G. boninense*, which presents information about its pathogenicity.

**Table 2:** Functional analysis of the secondary metabolites produced by the dikaryotic mycelia of *Ganoderma boninense.*

Pathway	Significant hits	− log10 *p-*value	FDR	Compound hits
Cysteine and methionine metabolism	3	1.003852	1.59E−01	S-adenosyl-l-methionine (C00019), S-adenosyl-l-homocysteine (C00021), l-homoserine (C00263)
Arginine and proline metabolism	3	1.003852	1.59E−01	l-Arginine (C00062), 4-guanidinobutanamide (C03078), S-adenosyl-l-methionine (C00021)
Phenylalanine metabolism	2	0.8606562	3.17E−02	Phenylpyruvate (C00166), phenylacetic acid (C07086)

Compound hits were based on Kyoto Encyclopedia of Genes and Genomes (KEGG) library. KEGG number is written in the parenthesis after the compound name.

## Discussion

In this study, the metabolic differences between the crude extracts of monokaryotic (monokaryon) and dikaryotic (dikaryon) mycelia of *Ganoderma boninense* grown in OPEM were investigated using LC–MS-based metabolomics to determine their pathogenicity. Both monokaryon and dikaryon extracts appeared to have a relatively higher number of OPEM-derived secondary metabolites than fungal metabolites. Monokaryon produced lesser fungal metabolites than dikaryon, suggesting that monokaryon had a lower possibility of inducing plant infection. These findings were further supported by the identified functional activities, which are involved in amino acid metabolism. The monokaryon exhibited tyrosine, phenylalanine and tryptophan metabolism, which are associated with fungal growth and development, as well as the synthesis of toxin precursors, thus rendering the monokaryon as non-infective. Dikaryon, on the other hand, is infective as it exhibited metabolism of cysteine and methionine, arginine and proline, and phenylalanine, which are essential in the virulence, pathogenicity, growth, and development of *G. boninense*. The findings of this study can be used as a model for understanding the pathogenesis of *G. boninense*, which can be integrated into disease management strategies to effectively control *Ganoderma* infections such as the basal stem rot (BSR). The identified functional activities of monokaryon can be used as targets to disrupt the key pathways associated with its growth and production of toxin precursors. Similarly, the identified functional activities of dikaryon that are associated with its virulence and pathogenicity can be disrupted to prevent plant infection and to serve as potential targets for the development of fungicides.

In this study, the crude extracts of monokaryon revealed the abundance of naturally occurring plant metabolites such as alkaloids, glycosides, hydroxybenzoic acids, flavonoids, lactones and fatty acids, which were also detected in the crude extracts of dikaryon^12^. As both fungi were grown in vitro in OPEM, these plant secondary metabolites may have originated from the oil palm and play a role in plant defense. For example, the alkaloid theobromine is a plant metabolite that acts as a plant defense against pathogens due to its toxicity^15,16^. Similarly, the plant metabolite p-salicylic acid was also detected in both monokaryon and dikaryon crude extracts. It is a hydroxybenzoic acid that is a known phytohormone that regulate plant immunity against biotic (e.g., microbial pathogens) and abiotic (e.g., heavy metals, salinity, ozone, ultraviolet, temperature and drought) stresses^17^, indicating its role in oil palm protection. Our findings suggest that both monokaryon and dikaryon trigger the synthesis of salicylic acid (i.e., induced synthesis) and that the oil palm responds to the presence of *Ganoderma* irrespective of the mycelial type through the production of salicylic acid.

Flavonoids and glycosides were also detected in monokaryotic and dikaryotic *G. boninense* crude extracts. These secondary metabolites were OPEM-derived and play crucial roles in protecting the oil palm from various stresses, including pathogen attack. Flavonoids are known to protect plants from various biotic and abiotic stresses and can also function as signaling molecules and defense substances^18,19^. Oil palms are also known to contain flavonoids that play a role in plant development and defense^20,21^. Similarly, glycosides are known to regulate plant growth and as part of the plant defense mechanism against herbivores and pathogens^22^. Furthermore, plant-derived long-chain fatty acids were also identified in this study. These fatty acids may be involved in plant defense mechanisms as signaling molecules^23^ and can also be used as carbon and energy sources for fungi^24^. These secondary metabolites originated from the oil palm, which supported the growth and development of *G. boninense.* These metabolites, however, are not involved in pathogenicity and virulence of *G. boninense*.

In this study, a low number of fungal metabolites was detected in both monokaryon and dikaryon crude extracts. For the monokaryon, the only fungal secondary metabolite detected is putatively identified as 14-dihydroxycornestin. In this study, the monokaryotic *G. boninense* produced 14-dihydroxycornestin to protect the oil palm from weeds and other weed pests, thereby ensuring that none may interfere with the host plant physiological processes that are beneficial to *G. boninense.* The metabolite 14-dihydroxycornestin is a cyclic dicarboxylic anhydride that is known to exhibit herbicidal activities against different weeds^25^. Weeds and weed pests are one of the most serious issues in agriculture and food safety as these actively compete with the growth of agricultural crops^26,27^. Interestingly, fungal plant pathogens are known to produce phytotoxins, which are secondary metabolites with unique chemical structures that induce disease symptoms in other plants and weeds^27^. As such, 14-dihydroxycornestin is beneficial to both oil palm and *G. boninense* as it serves as a protection against other organisms that may colonize or infect the oil palm. The metabolite 14-dihydroxycornestin, however, was not detected in the crude extracts of dikaryotic *G. boninense*. This indicates that the production of 14-dihydroxycornestin by the monokaryotic *G. boninense* is a protective strategy to eliminate other competing organisms and not to infect the oil palm. Other fungal metabolites such as aspulvinone H, bergenin and methylisocitric acid were detected in dikaryon crude extracts, which may play essential roles in the virulence and pathogenicity of *G. boninense*^12^.

The functional activities were also determined to correlate all the extracted secondary metabolites of both monokaryotic and dikaryotic *G. boninense* with their pathogenicity profiles. In this study, all the identified functional activities are involved in amino acid metabolism. Many pathogenic fungi can readily assimilate amino acids since carbon and nitrogen sources are abundant in host cells^28^. The functional activities of monokaryon included the metabolism of tyrosine, phenylalanine and tryptophan. Tyrosine metabolism provides the fungus with carbon and nitrogen sources for its growth^28^. Oil palm contains cellulose, hemicellulose and lignin, which are broken down by *G. boninense* through the release of enzymes^29^. The resulting lignin-derived monomers can then be metabolized by *G. boninense* to obtain carbon and nitrogen^30^. In this study, the identified significant metabolites in tyrosine metabolism are lignin-derived monomers and are thus essential for fungal growth. Furthermore, the metabolism of phenylalanine, tyrosine and tryptophan (also called as the three aromatic amino acids) is involved in fungal growth and are known precursors for several fungal downstream products such as mycotoxins^31^. These amino acids can be synthesized and utilized by fungi to yield potentially damaging mycotoxins^31^. In this study, the identified metabolic intermediates such as phenylacetate, phenylpyruvate, homovanillate, indoleglycerol phosphate and 3-methoxy-4-hydroxyphenylglycoaldehyde were necessary to metabolize the three aromatic amino acids in preparation for mycotoxin synthesis. As such, our findings suggest that monokaryon produces secondary metabolites that are essential to fungal growth and development as well as precursors to produce mycotoxins. At the monokaryotic stage, *G. boninense* is unable to produce mycotoxins and therefore considered non-pathogenic.

The functional activities of dikaryotic *G. boninense* also involved amino acid metabolism, which includes the metabolism of cysteine and methionine, arginine and proline and phenylalanine. In this study, the metabolic intermediates such as S-adenosyl-l-methionine, S-adenosyl-l-homocysteine and l-homoserine were enriched to synthesize cysteine and methionine. Cysteine and methionine metabolism is involved in the development and virulence of the fungus^32^. Sulfur-containing amino acids such as methionine and cysteine play important roles in cell proliferation, metabolism, protein synthesis, DNA methylation, multiple stress resistance and pathogenicity^32^. Deletion of certain genes that are involved in the metabolism of cysteine and methionine led to reduced fungal virulence and pathogenicity^32,33^, thereby proving the significance of these amino acids in fungi. Additionally, the metabolism of arginine and proline is identified in dikaryon. In this study, the metabolite l-arginine was identified and represented arginine metabolism, which is then further processed to metabolize proline. These amino acids are involved in fungal growth and pathogenicity as well as nitrogen metabolism and protein synthesis in fungi^34,35^. Finally, the metabolism of phenylalanine is also identified in dikaryon. Similar with monokaryon*,* phenylalanine metabolism plays an important role in fungal growth and development^31^. The ability of a pathogen to utilize amino acids as carbon and nitrogen sources for its growth is critical during its pathogenesis^36^. In this study, the metabolism of phenylalanine and proline supported the growth of *G. boninense* while metabolizing arginine, cysteine and methionine that are responsible for its pathogenicity. As such, our findings suggest that dikaryon produces secondary metabolites that are essential for its virulence, pathogenicity, growth and development. At this stage, *G. boninense* could produce mycotoxins, thereby rendering dikaryon pathogenic.

## Conclusions

This study revealed the differences between the metabolic profiles of monokaryotic (monokaryon) and dikaryotic (dikaryon) *G. boninense*, which were used to identify their functional activities that could be correlated with its pathogenicity. The crude extracts of monokaryon and dikaryon both contained abundant plant-derived secondary metabolites, which originated from OPEM. However, monokaryon produced lesser fungal metabolites than dikaryon. The functional activities revealed that *G. boninense* in the monokaryotic stage is unable to produce mycotoxins and is therefore considered non-pathogenic, whereas the dikaryon could produce mycotoxins as well as other secondary metabolites essential for its growth, thereby rendering it pathogenic. The LC–MS-based metabolomics approach and *mummichog* analyses of the crude extracts of *G. boninense* are thus effective tools to determine fungal pathogenicity. The results of this study could help devise new ways to intervene in the infection process of *G. boninense* (e.g., disrupting the functional activities that are associated with the growth and pathogenicity of the fungus) and therefore help protect the oil palm. The identified functional activities and their respective secondary metabolites may direct to specific genes that produce and express them, suggesting new targets for effective antifungals. A similar approach could be carried out to understand the pathogenesis of other economically important plant fungal pathogens of different crops, which can help control fungal pathogens effectively.

### Supplementary Information

Supplementary Table S1.

## Data Availability

The data generated during and/or analysed during this study are included in this published article (and its Supplementary Information files). The metabolomics datasets generated during and/or analysed during the current study are available from the corresponding author on reasonable request.
