# Supplementary material for: Pathogenicity of monokaryotic and dikaryotic mycelia of Ganoderma boninense revealed via LC–MS-based metabolomics
Source: Sci Rep. 2024 Mar 4;14:5330. doi: 10.1038/s41598-024-56129-8 (PMC10912678; doi:10.1038/s41598-024-56129-8)
Supplement: Supplementary file 1 — Supplementary Table S1. [file 41598_2024_56129_MOESM1_ESM.docx]

**Pathogenicity of monokaryotic and dikaryotic mycelia of *Ganoderma boninense* revealed via LC-MS-based metabolomics**

Krystle Angelique A. Santiago^1^, Wei Chee Wong^2^, You Keng Goh^2^, Seng Heng Tey^2^ and Adeline Su Yien Ting^1*^

^1^School of Science, Monash University Malaysia, Jalan Lagoon Selatan 47500 Bandar Sunway, Selangor Darul Ehsan, Malaysia

^2^Advanced Agriecological Research Sdn. Bhd., 11 Jalan Teknologi 3/6, Taman Sains Selangor 1, Kota Damansara, 47810 Petaling Jaya, Selangor Darul Ehsan, Malaysia

**Table S1** Secondary metabolites detected from the monokaryotic *Ganoderma boninense* (mGb) grown in oil palm extract medium (OPEM) broth and incubated at different time points (Day 1, 3, 7, 14, 21 and 28) using LC-Q/TOF analysis (negative ionization). Identification of these metabolites was based on the METLIN database. The list of secondary metabolites produced by dikaryotic *G. boninense* (OPEM + dGb) has been reported (Santiago et al. 2023).

| **Sample** | **Chemical Formula** | **Mass** | **Retention Time (min)** | **Putative Identification** | **Type of Metabolite (Classification)** | **Detected in OPEM + dGb** |
| --- | --- | --- | --- | --- | --- | --- |
| Day 1  (OPEM + mGB) | C_7_H_8_N_4_O_2_ | [M-H]^-^ 180.0645 | 0.652 | Theobromine | Plant Metabolite (alkaloid) | No |
|  | C_7_H_6_O_3_ | [M-H]^-^ 138.0319 | 6.533 | p-salicylic acid | Plant Metabolite (hydroxybenzoic acid) | Yes |
|  | C_20_H_20_O_10_ | [M-H]^-^ 420.1058 | 8.982 | 5,7,3′-trihydroxy-3,6,8,4′,5′-pentamethoxyflavone | Plant Metabolite (flavonoid) | Yes |
|  | C_17_H_22_O_5_ | [M+Cl]^-^ 306.1468 | 9.644 | Ligulatin B | Plant Metabolite (sesquiterpene lactone) | No |
|  | C_18_H_32_O_5_ | [M-H]^-^ 328.2259 | 11.099 | 11-hydroperoxy-12,13-epoxy-9-octadecenoic acid | Plant Metabolite (long-chain fatty acid) | Yes |
| Day 3  (OPEM + mGb) | C_7_H_8_N_4_O_2_ | [M-H]^-^ 180.0643 | 0.651 | Theobromine | Plant Metabolite (alkaloid) | No |
|  | C_7_H_6_O_3_ | [M-H]^-^ 138.0321 | 6.504 | p-salicylic acid | Plant Metabolite (hydroxybenzoic acid) | Yes |
|  | C_20_H_20_O_10_ | [M-H]^-^ 420.1067 | 8.982 | 5,7,3′-trihydroxy-3,6,8,4′,5′-pentamethoxyflavone | Plant Metabolite (flavonoid) | Yes |
|  | C_17_H_22_O_5_ | [M+Cl]^-^ 306.1467 | 9.648 | Ligulatin B | Plant Metabolite (sesquiterpene lactone) | No |
|  | C_18_H_32_O_5_ | [M-H]^-^ 328.2257 | 11.107 | 11-hydroperoxy-12,13-epoxy-9-octadecenoic acid | Plant Metabolite (long-chain fatty acid) | Yes |
|  | C_18_H_34_O_5_ | [M-H]^-^ 330.2414 | 12.138 | 5,8,12-trihydroxy-9-octadecenoic acid | Plant Metabolite (long-chain fatty acid) | Yes |
| Day 7  (OPEM + mGb) | C_7_H_6_O_3_ | [M-H]^-^ 138.0321 | 6.504 | p-salicylic acid | Plant Metabolite (hydroxybenzoic acid) | Yes |
|  | C_22_H_24_O_12_ | [M-H]^-^ 480.1248 | 8.915 | Torachrysone 8-(6-oxalylglucoside) | Plant Metabolite (glycoside) | Yes |
|  | C_17_H_22_O_5_ | [M+Cl]^-^ 306.1471 | 9.809 | Ligulatin B | Plant Metabolite (sesquiterpene lactone) | No |
|  | C_15_H_10_N_2_O_2_ | [M-H]^-^ 250.0747 | 9.808 | 5-methoxycanthin-6-one | Plant Metabolite (alkaloid) | No |
|  | C_18_H_32_O_5_ | [M-H]^-^ 328.2259 | 11.099 | 11-hydroperoxy-12,13-epoxy-9-octadecenoic acid | Plant Metabolite (long-chain fatty acid) | Yes |
|  | C_18_H_34_O_5_ | [M-H]^-^ 330.2414 | 12.138 | 5,8,12-trihydroxy-9-octadecenoic acid | Plant Metabolite (long-chain fatty acid) | Yes |
| Day 14  (OPEM + mGb) | C_7_H_8_N_4_O_2_ | [M-H]^-^ 180.0647 | 0.652 | Theobromine | Plant Metabolite (alkaloid) | No |
|  | C_16_H_18_O_10_ | [M-H]^-^ 370.0909 | 7.728 | Ferulic acid 4-O-glucuronide | Plant Metabolite (glycoside) | No |
|  | C_17_H_22_O_5_ | [M+Cl]^-^ 306.1468 | 9.645 | Ligulatin B | Plant Metabolite (sesquiterpene lactone) | No |
|  | C_26_H_26_O_6_ | [M-H]^-^ 434.1707 | 9.776 | Scandenin | Plant Metabolite (isoflavonoid) | No |
| Day 21  (OPEM + mGb) | C_7_H_6_O_3_ | [M-H]^-^ 138.032 | 6.545 | p-salicylic acid | Plant Metabolite (hydroxybenzoic acid) | No |
|  | C_22_H_24_O_12_ | [M-H]^-^ 480.1281 | 8.890 | Torachrysone 8-(6-oxalylglucoside) | Plant Metabolite (glycoside) | No |
|  | C_17_H_22_O_5_ | [M+Cl]^-^ 306.1468 | 9.643 | Ligulatin B | Plant Metabolite (sesquiterpene lactone) | No |
| Day 28  (OPEM + mGb) | C_22_H_24_O_12_ | [M-H]^-^ 480.1276 | 8.889 | Torachrysone 8-(6-oxalylglucoside) | Plant Metabolite (glycoside) | No |
|  |  |  |  | Dihydrophaseic acid 4-O-beta-D-glucoside | Plant Metabolite (beta-D-glucoside) | No |
|  | C_17_H_22_O_5_ | [M+Cl]^-^ 306.1468 | 9.647 | Ligulatin B | Plant Metabolite (sesquiterpene lactone) | No |
|  | C_30_H_34_O_8_ | [M-H]^-^ 522.2226 | 10.555 | Carpelastofuran | Plant Metabolite  (flavone) | No |
|  | C_15_H_10_N_2_O_2_ | [M-H]^-^ 250.0744 | 11.098 | 5-methoxycanthin-6-one | Plant Metabolite (alkaloid) | No |
|  | C_16_H_20_O_6_ | [M-H]^-^ 308.1267 | 11.596 | 14-dihydroxycornestin | Fungal Metabolite  (cyclic dicarboxylic anhydride) | No |
